# Sex differences in procedural characteristics, safety, and clinical outcomes of pulsed field ablation for atrial fibrillation

**DOI:** 10.1016/j.hroo.2025.10.010

**Published:** 2025-10-24

**Authors:** Fabian Jordan, Behnam Subin, Corinne Isenegger, Jonas Brügger, Jeanne du Fay de Lavallaz, Christine S. Zuern, Emel Kaplan, David Spreen, Sven Knecht, Philipp Krisai, Nicolas Schaerli, Beat Schär, Gian Völlmin, Felix Mahfoud, Christian Sticherling, Michael Kühne, Patrick Badertscher

**Affiliations:** 1 Department of Cardiology, University Hospital Basel, Basel, Switzerland; 2 Cardiovascular Research Institute Basel, University Hospital Basel, Basel, Switzerland; 3 Department of Rhythmology, University Hospital Schleswig-Holstein, Lübeck, Germany

**Keywords:** Atrial fibrillation, Pulsed field ablation, Pulmonary vein isolation, Sex differences, Procedural characteristics, Complications, Outcomes, 1-year follow-up

## Abstract

**Background:**

Previous studies on pulmonary vein isolation (PVI) for the treatment of atrial fibrillation (AF) using conventional thermal techniques have shown inconsistent sex-related outcomes. Pulsed field ablation (PFA) is a novel energy source offering myocardial-selective ablation. However, sex-specific data on its performance are limited.

**Objective:**

This study aimed to compare procedural characteristics, safety, and clinical outcomes of PFA in female and male patients undergoing AF ablation.

**Methods:**

This prospective study included consecutive patients with paroxysmal or persistent AF undergoing PFA-based PVI using a pentaspline catheter. Follow-up was conducted at 3, 6, and 12 months. A 1:1 propensity score-matched cohort was created to evaluate sex-specific outcomes.

**Results:**

Among 425 patients, 134 (32%) were women. Compared with men, women were older (median 70 vs 65 years; *P* < .001), more frequently had paroxysmal AF (60% vs 45%; *P* = .006), and had lower rates of coronary artery disease (6% vs 14%; *P* = .029). Although overall procedural times were similar, female patients with paroxysmal AF had significantly longer procedure, left atrial dwell, and fluoroscopy times than males. Complication rates were comparable (1.5% in women vs 0.7% in men; *P* = .371). After propensity score matching (133 women to 133 men), arrhythmia recurrence at 1-year follow-up was higher in women (23% vs 12%; *P* = .017; hazard ratio 2.2; standard error 0.34).

**Conclusion:**

Significant sex-related differences exist in clinical outcomes after PVI with PFA in AF. Further studies exploring underlying mechanisms and tailored approaches may enhance outcomes in female patients.

## Introduction

The impact of sex on the risks and benefits of catheter ablation for atrial fibrillation (AF) remains a subject of ongoing debate.^1^^,^^2^ Previous studies evaluating sex-specific disparities in clinical outcomes after conventional thermal ablation modalities—such as radiofrequency or cryoablation—have reported worse long-term efficacy and higher short-term complication rates in female patients than male patients.^2^, ^3^, ^4^, ^5^, ^6^, ^7^, ^8^, ^9^, ^10^, ^11^, ^12^, ^13^

Pulsed field ablation (PFA) is a novel nonthermal ablation modality that uses irreversible electroporation to achieve selective myocardial tissue ablation.^14^, ^15^, ^16^, ^17^, ^18^, ^19^, ^20^, ^21^, ^22^, ^23^, ^24^ Preclinical studies have demonstrated its specificity for cardiac tissue, whereas clinical trials have confirmed its favorable safety profile^25^, ^26^, ^27^, ^28^, ^29^ and reported variable efficacy rates ranging from 55% to 92%,^29^^,^^30^^,^^31^ yet few studies have specifically examined sex disparities after PFA.

PFA may help mitigate sex-related differences in AF ablation outcomes for several reasons. First, PFA preferentially affects myocardium and thus may lessen technique-related constraints linked to atrial wall thickness that can complicate thermal ablation; this potential advantage is safety and consistency related rather than depth of lesion related.^32^ Second, the tissue-selectivity of PFA may reduce collateral injury to adjacent structures such as the esophagus, phrenic nerve, and pulmonary veins—sites where complications have historically been more common in female patients owing to anatomic differences. Third, the rapid and predictable energy delivery of PFA may reduce procedure times and technical complexity compared with thermal ablation techniques, particularly in patients with smaller left atria (LAs) or more advanced atrial fibrosis, both of which are more prevalent in female patients.^6^^,^^30^^,^^31^^,^^33^

Taken together, these features suggest that PFA may provide a safer and more consistent approach to AF ablation and hold promise in reducing sex-based disparities in clinical outcomes. In this study, we aimed to test this hypothesis by comparing procedural characteristics, safety, and clinical outcomes of PFA in female and male patients undergoing AF ablation.

## Methods

### Patient population

Consecutive patients who were referred to a tertiary referral center for pulmonary vein isolation (PVI) and were treated using the pentaspline PFA catheter (FARAPULSE, Boston Scientific Inc, Marlborough, MA) between January 2022 and July 2024 were prospectively enrolled in this study. Only patients undergoing a first AF ablation procedure were enrolled. All patients provided a written informed consent, and all patient information was anonymized. The study was approved by the local ethics board (SWISS-AF-PVI registry, NCT03718364) and performed in accordance with the ethical standards laid down in the 1964 Declaration of Helsinki and its later amendments.

### Procedures

#### Preprocedural imaging

All patients underwent preprocedural transesophageal echocardiography to rule out LA thrombus. In addition, all patients underwent preprocedural imaging with either computed tomography or magnetic resonance imaging of the LA.

#### Intraprocedural management

The procedure was performed under deep sedation using midazolam, fentanyl, and propofol. Femoral access was obtained using ultrasound guidance in all cases. A diagnostic catheter was introduced and positioned inside the coronary sinus. The transseptal puncture was performed via fluoroscopic guidance or in selected cases using intracardiac echocardiography. Intravenous heparin was used to keep the activated clotting time at a target value of 350 seconds. The intracardiac electrograms and surface electrograms were recorded at a speed of 100 mm/s (Sensis, Siemens, Erlangen, Germany).

#### PFA

A detailed description of our procedural workflow using the FARAPULSE PFA system has been described previously.^34^ In brief, after transeptal puncture, a J-tip guidewire was used to cannulate the PV, and the device was deployed inside the LA. The PFA procedure was performed based on a standard protocol^35^^,^^36^ with 4 applications in a spherical “basket” configuration and 4 applications in a fully deployed “flower” configuration per vein. To ensure uniform coverage across the entire circumference, a rotation of the catheter by 30–40° was performed after 2 successive applications in each configuration. Additional lesions for the right-sided carina were applied as previously described.^34^ Ablations were performed using 2 kV.

The procedural end point was defined as acute PVI, assessed directly at the end of the procedure for all PV. For end point confirmation, entrance and, if deemed necessary, exit blocks were tested via the PFA catheter by pacing with 10 V and a pulse width of 1.5 ms among all electrodes or via a 3-dimensional electroanatomic mapping (EAM) system (CARTO 3, Biosense Webster, Irvine, CA).

#### Postprocedural management

Hemostasis was established using figure-of-8 sutures, followed by 4 hours of bed rest. Pericardial effusion was ruled out by transthoracic echocardiography within 1 hour after intervention. Oral anticoagulation was continued for at least 2 months after ablation. In patients with persistent AF who had previously tolerated antiarrhythmic medication, therapy was generally continued for 2–3 months. Conversely, in patients with paroxysmal AF or who experienced adverse effects from antiarrhythmic drugs, the medication was discontinued at discharge.

#### Biomarkers of myocardial injury

Blood samples were collected in a fasting state on the morning before the procedure and 24 hours after the procedure. A high-sensitivity cardiac troponin T assay (Roche Elecsys 2010 high-sensitivity troponin T; Roche Diagnostics, Indianapolis, IN) with a 99th percentile concentration of 14 ng/L with a corresponding coefficient of variation of 10% at 13 ng/L was used.^37^

#### Follow-up

The primary efficacy end point of the study was freedom from any atrial arrhythmia recurrence during a 1-year follow-up period. After a 2-month blanking period, any episode of AF, atrial flutter, or atrial tachycardia lasting more than 30 seconds was considered a recurrence.^38^ If an arrhythmia first occurred during the blanking period and persisted beyond it, the earliest onset of the arrhythmia was recorded as the time of recurrence.

Secondary (efficiency) end points included total procedural duration (from groin puncture to sheath removal), LA dwell time, and fluoroscopic time. Safety end points comprised phrenic nerve injury, pericardial tamponade, transient ischemic attack or stroke, and vascular access complications.

Follow-up assessments, including physical examination, 12-lead electrocardiogram, and 7-day Holter monitoring, were conducted at 3, 6, and 12 months. The same follow-up schedule applied to patients with cardiovascular implantable electronic devices, except for those with implantable loop recorders, who underwent remote monitoring for arrhythmia detection. In the event of symptomatic recurrence outside the scheduled follow-up visits, arrhythmia was documented by either a 12-lead electrocardiogram or Holter recording.

### Statistical analysis

Continuous variables are described using median and interquartile range (IQR) and compared using the Wilcoxon rank sum test. Categorical variables are presented as numbers and percentages and compared using χ^2^ or Fisher’s exact test, as appropriate.

The Kaplan-Meier analysis with a log-rank test was used to compare the probability of freedom from the late recurrence of atrial arrhythmia. *P* < .05 was considered statistically significant.

For the regression models, age was not included in the CHA_2_DS_2_-VA score to prevent interaction between age and CHA_2_DS_2_-VA score in the multivariate regression models, henceforth called “CHA_2_DS_2_-VA minus age.”

Multivariate regression was performed to identify predictors of late recurrence at 1-year follow-up. Potential confounders were entered into the model based on known or expected clinical relevance, regardless of statistically significant differences between groups stratified by arrhythmia recurrence. Variables included in the model were age, body mass index (BMI), sex, CHA_2_DS_2_-VA minus age, AF type, LA diameter, history of heart failure, and whether additional lesions were performed. Odds ratios (ORs) along with 95% confidence intervals (CIs) from the regression model are reported in the results.

Nearest neighbor propensity score matching (ratio 1:1, female to male) without replacement with a propensity score estimated using logistic regression of sex on the covariates (age, BMI, LA, persistent AF, hypertension, stroke, diabetes, heart failure, and myocardial infarction) was performed.

Analysis was performed using R (R Core Team [2021], R Foundation for Statistical Computing, Vienna, Austria) and RStudio 2023.09.1 (RStudio Team [2019], RStudio, Inc, Boston, MA).

## Results

### Baseline characteristics

A total of 425 patients (median [IQR] age 67 [60–73] years; 49.4% paroxysmal AF) underwent a first PVI using a pentaspline PFA system. Of these, 134 patients were women (32%) and 291 were men (68%). Compared with male patients, female patients were significantly older (median [IQR] age 70 [62–75] years vs 65 [58–72] years; *P* < .001), more frequently diagnosed as having paroxysmal AF (59.7% [80 of 134] vs 44.7% [130 of 291]; *P* = .006), and less commonly affected by coronary heart disease (6.0% [8 of 134] vs 13.7% [40 of 291]; *P* = .029). There was no statistically significant difference in the use of antiarrhythmic drugs between the 2 groups. The baseline characteristics between male and female individuals are presented in Table 1.

**Table 1:** Baseline characteristics

	Overall	Women	Men	*P* value
n	425	134	291	
Age, y	67 [60–73]	70 [62–75]	65 [58–72]	<.001
BMI, kg/m^2^	26.6 [23.9–29.6]	25.2 [22.8–29.4]	27.1 [24.5–29.7]	.011
Paroxysmal AF	210 (49.4)	80 (59.7)	130 (44.7)	.006
Persistent AF	207 (48.7)	51 (38.1)	156 (53.8)	.004
Diabetes	44 (10.4)	13 (9.7)	31 (10.7)	.898
Hypertension	249 (58.6)	77 (57.5)	172 (59.1)	.831
CAD	48 (11.3)	8 (6.0)	40 (13.7)	.029
Heart failure	68 (16.0)	24 (17.9)	44 (15.1)	.557
Stroke	11 (2.6)	3 (2.2)	8 (2.7)	1
LA	41 [36–45]	39 [35–43]	42 [38–47]	<.001
LAVI, mL/m^2^	38 [30–46]	39 [29–47]	38 [30–46]	.92
LVEF, %	57 [50–62]	60 [53–64]	56 [50–61]	.001
Septum thickness, mm	10 [9–11]	9 [8–10]	10 [9–12]	<.001
Posterior wall thickness, mm	9 [8–11]	9 [7–10]	9 [9–11]	<.001
AAD	171 (40.2)	58 (43.3)	113 (38.8)	.445
Betablocker	305 (71.8)	102 (76.1)	203 (69.8)	.216
Vitamin K antagonist (ie, Marcoumar)	16 (3.8)	6 (4.5)	10 (3.4)	.898
DOAC	364 (85.6)	118 (88.1)	246 (84.5)	.531
ILR	42 (9.9)	8 (6.0)	34 (11.7)	.097

Continuous variables expressed as “median [IQR]” and factorial variables as “absolute number (%).”

AAD = antiarrhythmic drug; AF = atrial fibrillation; BMI = body mass index; CAD = coronary artery disease; DOAC =direct oral anticoagulation; ILR = implantable loop recorder; IQR = interquartile range; LA = left atrium; LAVI = left atrial volume index; LVEF = left ventricular ejection fraction.

### Procedural characteristics

Median procedure time, LA dwell time, and fluoroscopic time were similar in female and male patients (Table 2), whereas, in patients with paroxysmal AF (n = 210; 49.4%), procedure times (54 [40–68] vs 40 [29–56] minutes; *P* < .001), LA dwell times (36 [23–53] vs 24 [16–37] minutes; *P* < .001), and fluoroscopy times (12 [8–14] vs 9 [7–13] minutes; *P* = .003) were significantly longer in female than male patients. In addition, EAM was more frequently used in female patients (56.2% vs 38.5%; *P* = .018) (Supplemental Table 1). In patients with persistent AF (n = 207; 48.7%), female patients had similar procedure times, LA dwell times, and fluoroscopy times, but lower fluoroscopy doses than male patients (377 [148–618] vs 538 [289–957] μGy∗m^2^; *P* = .001). In addition, EAM was less frequently used in female patients (62.7% vs 78.2%; *P* = .044) (Supplemental Table 2; for comparison of procedural characteristics between paroxysmal and persistent AF, see Supplemental Table 3).

**Table 2:** Procedural characteristics

	Overall	Women	Men	*P* value
n	425	134	291	
Procedure time, min	50 [38–66]	51 [40–67]	50 [36–65]	.349
LA time, min	35 [22–49]	36 [23–50]	35 [22–48]	.498
Fluoroscopy time, min	10 [8–13]	11 [8–14]	10 [8–13]	.011
Fluoroscopy dose, μGy∗m^2^	387.0 [228.5–792.5]	345.0 [149.0–645.0]	415.0 [247.2–910.5]	.001
Total applications	34 [28–50]	34 [26–50]	34 [28–50]	.197
PVI applications	32 [18–34]	32 [18–34]	32 [19–34]	.328
Catheter size, 35 mm	32 (7.5)	9 (6.7)	23 (7.9)	.816
Additional lesions	137 (32.2)	41 (30.6)	96 (33.0)	.705
Mapping	254 (59.8)	78 (58.2)	176 (60.5)	.736
Hs-cTnT after PVI, ng/L	1412 [946–1906]	1369 [972–2138]	1420 [931–1844]	.335

Continuous variables expressed as “median [IQR]” and factorial variables as “absolute number (%).”

Hs-cTnT = high-sensitivity cardiac troponin T; IQR = interquartile range; LA = left atrial; PVI = pulmonary vein isolation.

PVI was successfully achieved in all patients. The number of applications was 34 [26–50] for female and 34 [28–50] for male patients (*P* = .197). Additional ablation beyond PVI was performed in 30.6% of female (41 of 134) and 33.0% of male patients (96 of 291; *P* = .705). Periprocedural mapping was performed in 58.2% of female and 60.5% of male patients. When comparing only patients who received PVI without EAM (Supplemental Table 4) and patients who received PVI with the use of EAM (Supplemental Table 5), findings of procedural characteristics for female and male patients were mirrored. There were no differences detected regarding PV anomalies such as left common ostium or right-sided middle PV between the groups (20.9% vs 20.6%; *P* = 1).

Additional ablation beyond PVI was performed in 30.6% of women and 33.0% of men (*P* = .705), consisting predominantly of right-sided carina reinforcement within the standardized pentaspline PFA workflow; posterior wall or other linear lesions were used infrequently and only in selected cases.

High-sensitivity cardiac troponin T as a marker of myocardial injury was measured the day after CA and was not statistically significantly higher in female vs male patients with 1369 ng/L [972–2138] vs 1420 ng/L [931–1844] (*P* = .335).

### Safety outcomes

Complications were rare (11 or 2.6% in the whole cohort) and comparable among the sexes (female 5 or 3.7% vs male 6 or 2.1%; *P* = .498). Cardiac tamponade occurred in 2 female patients (1.5%) and 1 male patient (0.3%); all of them were treated by drainage. There was 1 female patient with a stroke with no residual neurologic sequelae. No esophageal fistula or phrenic nerve damages were reported.

### Follow-up

**Figure 1:**
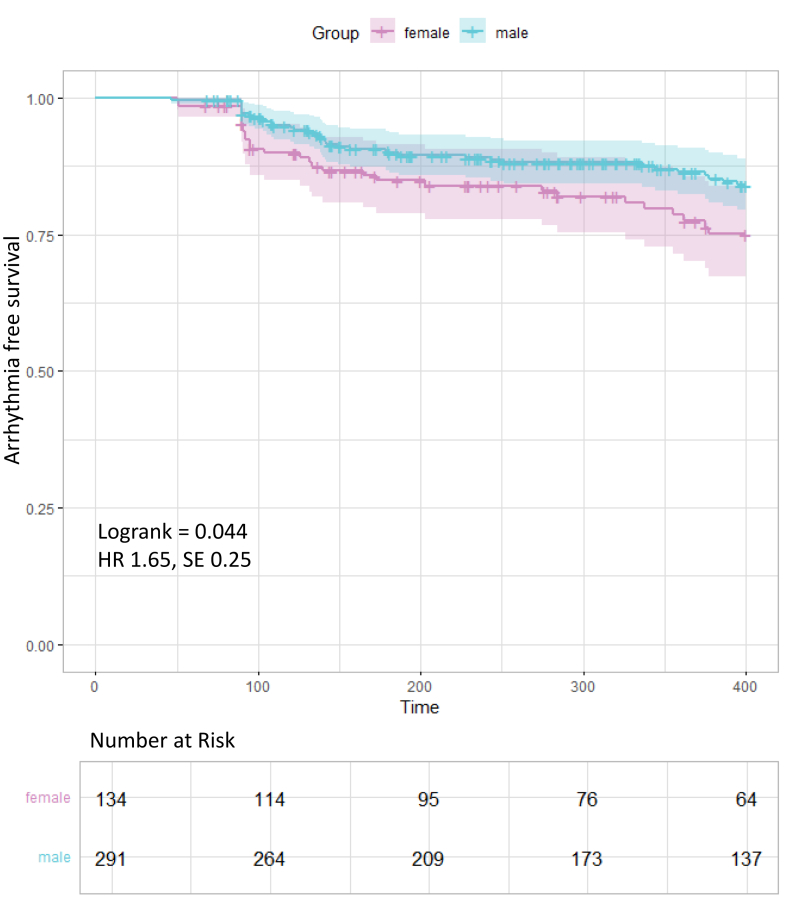
Kaplan-Meier curve comparing female and male patients in the overall population. The log-rank test was used to determine the *P* value. Time in days. HR, female to male. HR = hazard ratio; SE = standard error.

Median follow-up duration was 320 days [194.0–542.0] with no statistically significant differences between the 2 groups (348.5 [201.5–607.5] vs 314 [193.5–508] days; *P* = .23). The 1-year Kaplan-Meier estimate for freedom from AF, atrial flutter, and atrial tachycardia was lower in female than male patients (78% vs 85%; log-rank test *P* = .044; hazard ratio [HR] 1.65 [standard error {SE} 0.25]) (Figure 1). There was no significant difference in the median time to first AF recurrence between the sexes (female 133 [91–316] days vs male 142 [105–348] days; *P* = .337). When only including patients with paroxysmal AF, clinical efficacy was not statistically significantly different between female and male patients (79% vs 83%; log-rank test *P* = 0.422; HR 1.31 [SE 0.34]) (Supplemental Figure 1). When only including patients with persistent AF, female patients had lower arrhythmia-free survival than male patients (75% vs 87%; log-rank test *P* = .030; HR 2.24 [SE 0.37]) (Supplemental Figure 2; comparing paroxysmal to persistent AF, see Supplemental Figure 3). When only including patients without any additional lesions (PVI only), female patients had comparable arrhythmia-free survival with male patients (76% vs 83%; log-rank test *P* = .106; HR 1.59 [SE 0.29]) (Supplemental Figure 4).

Of the 75 patients with recurrence of atrial arrhythmia after the index PFA procedure, redo-procedure rates were comparable between the sexes (female 13 [43.3%] vs male 23 [51.1%]; *P* = .671) and the redo-procedure rates of durable PVI were comparable (female 7 (53.8%) vs male 13 [56.5%]; *P* = 1). The overall rate of hospitalizations during the follow-up period was low, but more frequent in female patients (female 8 or 6% vs male 5 or 1.7%; *P* = .03).

**Figure 2:**
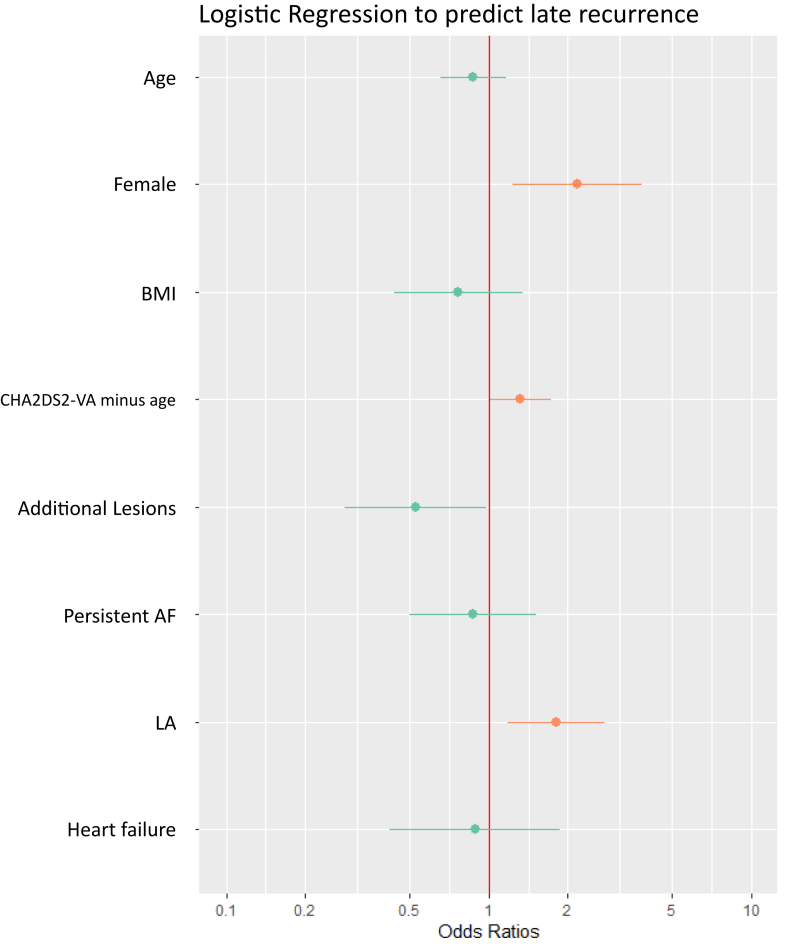
Forrest plot for the odds ratios from the logistic regression model fitted to identify potential predictors for atrial arrhythmia recurrence adjusted for the variables age (years), sex, BMI (kg/m^2^), CHA_2_DS_2_-VA score without age accounted for, whether additional lesions were performed (binary, yes/no), atrial fibrillation type (binary, levels: persistent/paroxysmal), LA diameter (mm), and history of heart failure (binary, yes/no). Odds ratios are presented in Supplemental Table 6. AF = atrial fibrillation; BMI = body mass index; LA = left atrial.

A multivariate Cox regression model to predict AF recurrence was fitted including the covariates age, female sex, BMI, CHA_2_DS_2_-VA minus age, additional lesions, persistent AF, LA, and heart failure. Female sex was the strongest statistically significant predictor with an OR of 2.16 (95% CI 1.23–3.81; *P* = .007) (Figure 2, Supplemental Table 6).

### Sensitivity analysis with a propensity score-matched population

The propensity-matched cohort included 266 patients (133 male, 133 female). The median age was 70 years [IQR 62–75], and the median BMI was 26.0 kg/m^2^ (IQR 23.5–29.4). The mean CHA_2_DS_2_-VA score was 1.98 (standard deviation 1.3). The population was matched for the covariates age, BMI, LA, persistent AF, history of arterial hypertension, stroke, myocardial infarction, coronary artery disease, diabetes, and heart failure. After matching, all standardized mean differences for the covariates were <0.2, indicating adequate balance. Other baseline characteristics were similar between sexes in the propensity-matched cohort (Supplemental Table 7). Procedural characteristics did not significantly differ in this propensity score-matched population.

In the propensity-matched cohorts, there was a statistically significant difference in freedom from atrial arrhythmia recurrence between sexes (77% vs 88%; log-rank test *P* = .017) (Supplemental Figure 5).

A multivariate Cox regression model to predict AF recurrence was also fitted for this cohort including the same covariates mentioned: age, female sex, BMI, CHA_2_DS_2_-VA minus age, additional lesions, persistent AF, LA, and heart failure. Female sex was the strongest statistically significant predictor with an OR of 2.28 (95% CI 1.16–4.48; *P* = .016) (Supplemental Figure 6, Supplemental Table 8).

## Discussion

In this prospective cohort study of patients undergoing PFA for the treatment of AF, we identified important sex-specific differences in procedural characteristics and clinical outcomes. Our main findings are as follows:

First, female patients were older than male patients but had fewer cardiovascular comorbidities. Second, overall procedural characteristics were comparable between sexes. However, in patients with paroxysmal AF, procedure duration and LA dwell time were longer in female patients. Third, the overall complication rate was low and did not differ between sexes. Fourth, female individuals experienced significantly higher rates of arrhythmia recurrence during 1-year follow-up, even in a propensity-matched cohort.

These findings expand upon previous literature by demonstrating that, even with a nonthermal, tissue-selective energy source such as PFA, sex-related disparities in AF ablation outcomes persist. Previous research on conventional thermal ablation modalities, including radiofrequency and cryoablation, has consistently reported that female patients are referred for ablation at older ages and tend to experience higher rates of both complications and arrhythmia recurrence than male patients.^1^, ^2^, ^3^, ^4^, ^5^^,^^8^, ^9^, ^10^, ^11^, ^12^, ^13^^,^^30^^,^^31^^,^^33^^,^^39^^,^^40^ Our results align with these observations: female patients were significantly older at the time of ablation, a pattern attributed to delayed diagnosis or referral and the later onset of AF in females owing to their lower baseline cardiovascular risk.^1^^,^^2^^,^^4^^,^^33^

Interestingly, our study showed that female patients had a lower prevalence of coronary artery disease and comparable rates of other comorbidities. These findings reinforce the notion that female patients may present with AF in the absence of overt structural heart disease,^5^^,^^9^^,^^10^^,^^30^^,^^31^ a factor that may influence response to ablation response.

Consistent with recent findings from Turagam et al,^39^ we observed no significant sex differences in overall procedural duration or complication rates with PFA. This confirms the reproducibility of PFA as a safe and efficient ablation strategy across sexes. However, when stratified by AF type, female patients with paroxysmal AF had longer procedure and dwell times, potentially reflecting greater procedural complexity owing to anatomic differences, more frequent use of EAM (56% vs 39%) owing to nonpulmonary vein triggers, or more extensive atrial fibrosis.

The most notable sex-based disparity in our study was the significantly higher rate of arrhythmia recurrence in female patients during follow-up, persisting after propensity score matching. These findings stand in contrast to Turagam et al^39^ but mirror earlier data on thermal ablation modalities^1^, ^2^, ^3^^,^^7^^,^^8^^,^^10^^,^^12^^,^^30^^,^^40^^,^^41^ and suggest that procedural uniformity alone is insufficient to overcome sex-based differences in ablation outcomes.

Several mechanisms may underlie the lower arrhythmia-free survival in female patients. First, anatomic differences—such as smaller LAs and thinner atrial walls—may complicate durable lesion formation even with electroporation. Second, female individuals may have a higher burden of nonpulmonary vein triggers.^6^^,^^30^^,^^31^^,^^33^^,^^42^^,^^43^ Third, delays in referral or underuse of early rhythm control strategies in female patients may lead to more advanced substrate at the time of intervention, contributing to poorer outcomes.

The most notable differences between our cohort and that of Turagam et al^39^ included a lower BMI, shorter LA diameter among women, and a higher prevalence of heart failure. Although these factors may have influenced the observed differences in outcomes, their exact contribution remains uncertain and warrants further investigation. Of note, among patients undergoing redo ablation procedures, the rates of durable PVI were similar between sexes, suggesting that acute lesion creation may not differ substantially.

Although the MANIFEST-PF registry^33^ included a large, multinational cohort with heterogeneous workflows and follow-up strategies, our prospective single-center study applied a uniform, protocolized pentaspline PFA approach and incorporated structured 7-day Holter monitoring at predefined intervals. These results suggest that sex-based disparities in ablation outcomes are not fully mitigated by electroporation and may become more apparent when systematic rhythm surveillance is implemented.

Our findings indicate that although PFA is a promising and safe technology, its current implementation does not fully resolve sex-based differences in clinical outcomes. In light of the higher 1-year recurrence observed in women in our cohort, most evident in persistent AF, several procedural and follow-up considerations may be helpful. After confirming durable PVI, a structured search for nonpulmonary vein triggers with pharmacologic provocation and pacing, followed by targeted ablation of reproducible foci, could be considered. In persistent AF, a substrate-informed strategy, using EAM where available or imaging surrogates, to guide selective adjunctive lesions (eg, reinforcement at carinae or limited posterior wall work) may be preferable to empirical lines. Careful adherence to a uniform pentaspline PFA workflow with complete circumferential and carina coverage may also reduce residual gaps. Where feasible, enhanced rhythm surveillance (eg, extended Holter or implantable monitoring) can facilitate earlier recognition of recurrence. These measures are intended as pragmatic options and warrant evaluation in prospective studies.

### Limitations

Several limitations must be acknowledged. First, this was a single-center study, which may limit generalizability. Second, although extensive propensity score matching and multivariable adjustment were performed, residual confounding by unmeasured factors cannot be excluded. Third, AF burden was not systematically assessed, given that monitoring was limited to intermittent Holter recordings. Thus, we cannot exclude that differences in recurrence rates by sex may not translate into comparable AF burden, as has been suggested by recent studies using continuous monitoring.^44^ Fourth, detailed atrial substrate characterization with EAM was not performed, limiting our ability to directly assess sex-related differences in atrial remodeling within this cohort… Nevertheless, the follow-up methods reflect routine clinical practice and are consistent with real-world detection of arrhythmia recurrence.^45^^,^^46^

## Conclusion

Despite the procedural efficiency and favorable safety profile of PFA, important sex differences persist in clinical outcomes after AF ablation. These findings underscore the need for sex-specific considerations in AF ablation strategies and suggest that further refinements in patient selection, procedural technique, and adjunctive therapies may be necessary to optimize outcomes in female patients.

## Disclosures

Patrick Badertscher has received research funding from the “University of Basel,” the “Stiftung für Herzschrittmacher und Elektrophysiologie,” the “Freiwillige Akademische Gesellschaft Basel,” the “Swiss Heart Foundation,” and Johnson & Johnson and reports personal fees from Bristol Myers Squibb (BMS), Boston Scientific, and Abbott, all outside the submitted work. Philipp Krisai reports speaker fees from BMS/Pfizer. Grants from the Swiss National Science Foundation, Swiss Heart Foundation, Foundation for Cardiovascular Research Basel, and Machaon Foundation. Sven Knecht has received funding from the “Swiss Heart Foundation.” Felix Mahfoud has been supported by Deutsche Forschungsgemeinschaft (SFB TRR219, project ID 322900939) and Deutsche Herzstiftung. Saarland University has received scientific support from Ablative Solutions, Medtronic, and ReCor Medical. Until May 2024, F.M. has received speaker honoraria/consulting fees from Ablative Solutions, AstraZeneca, Inari, Medtronic, Merck, Novartis, Philips, and ReCor Medical. Christian Sticherling is a member of Medtronic Advisory Board Europe and Boston Scientific Advisory Board Europe and received educational grants from Biosense Webster and Biotronik and a research grant from the European Union’s FP7 program and Biosense Webster and lecture and consulting fees from Abbott, Medtronic, Biosense Webster, Boston Scientific, MicroPort, and Biotronik, all outside the submitted work. Michael Kuehne reports grants from the Swiss National Science Foundation (grant numbers 33CS30_148474, 33CS30_177520, 32473B_176178, 32003B_197524), the Swiss Heart Foundation, the Foundation for Cardiovascular Research Basel, the University of Basel, Bayer, Pfizer, Boston Scientific, BMS, and Biotronik and grants and personal fees from Daiichi Sankyo, all outside the submitted work. Behnam Subin reports travel grants from Boston Scientific, all outside the submitted work. Christine S. Zuern reports speaker and consulting fees from AstraZeneca, Boehringer Ingelheim, Pfizer, Medtronic, and Vifor Pharma. Others have nothing to declare.
