# Supplementary material for: Sex differences in procedural characteristics, safety, and clinical outcomes of pulsed field ablation for atrial fibrillation
Source: Heart Rhythm O2. 2025 Oct 24;7(1):37–45. doi: 10.1016/j.hroo.2025.10.010 (PMC12902224; doi:10.1016/j.hroo.2025.10.010)
Supplement: Supplement Figure 3 [file mmc3.pdf]

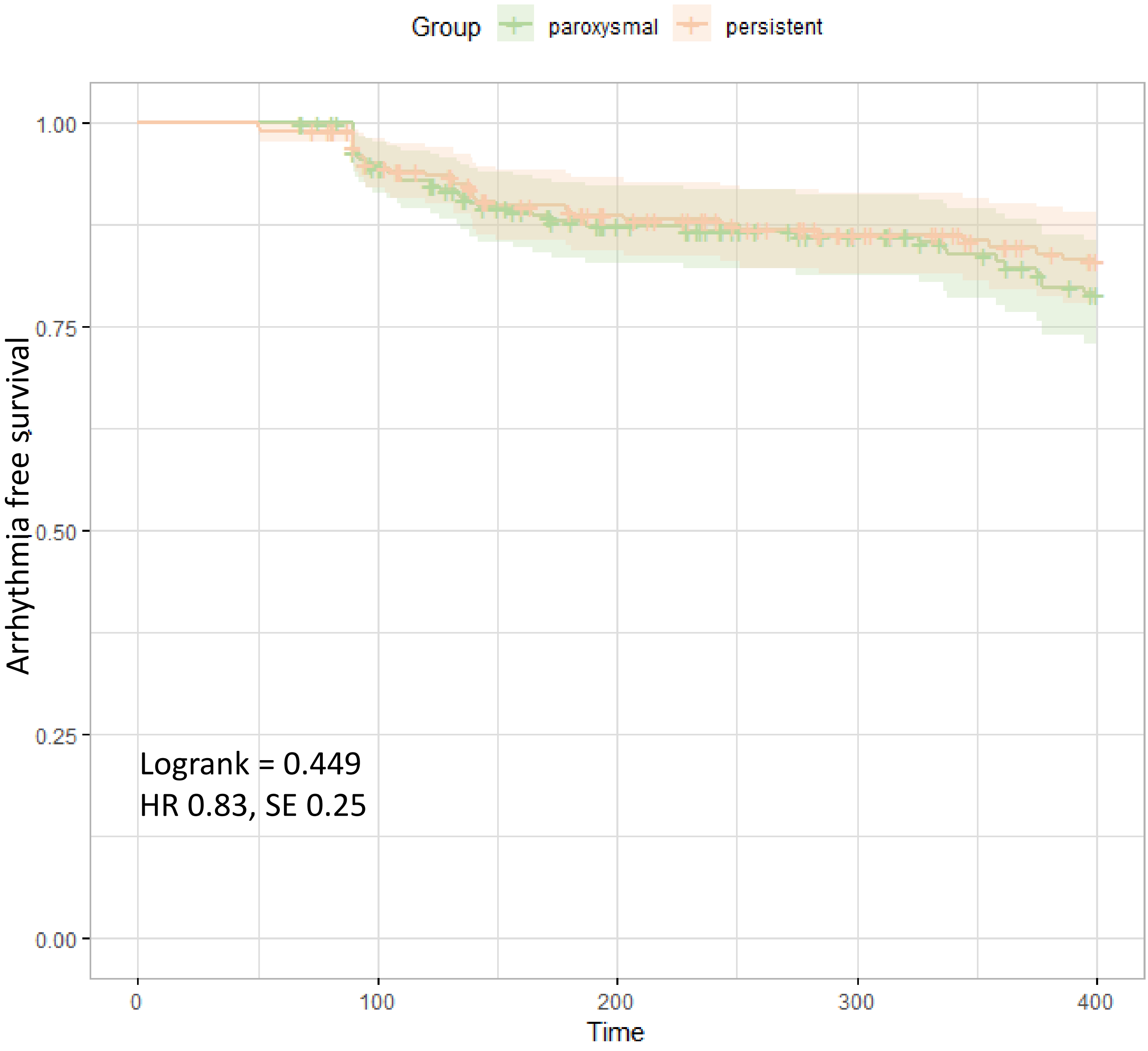

Number at Risk

paroxysmal	210	183	145	120	97
persistent	207	189	155	125	102

Supplement Figure 3: Kaplan Meier curve comparing patients with paroxysmal atrial fibrillation vs persistent atrial fibrillation in the overall population. The log rank test was used to determine the p-value. Time in days. Hazard Ratio (HR) persistent to paroxysmal, Standard error (SE).
