# Supplementary material for: Sex differences in procedural characteristics, safety, and clinical outcomes of pulsed field ablation for atrial fibrillation
Source: Heart Rhythm O2. 2025 Oct 24;7(1):37–45. doi: 10.1016/j.hroo.2025.10.010 (PMC12902224; doi:10.1016/j.hroo.2025.10.010)
Supplement: Supplement Figure 5 [file mmc5.pdf]

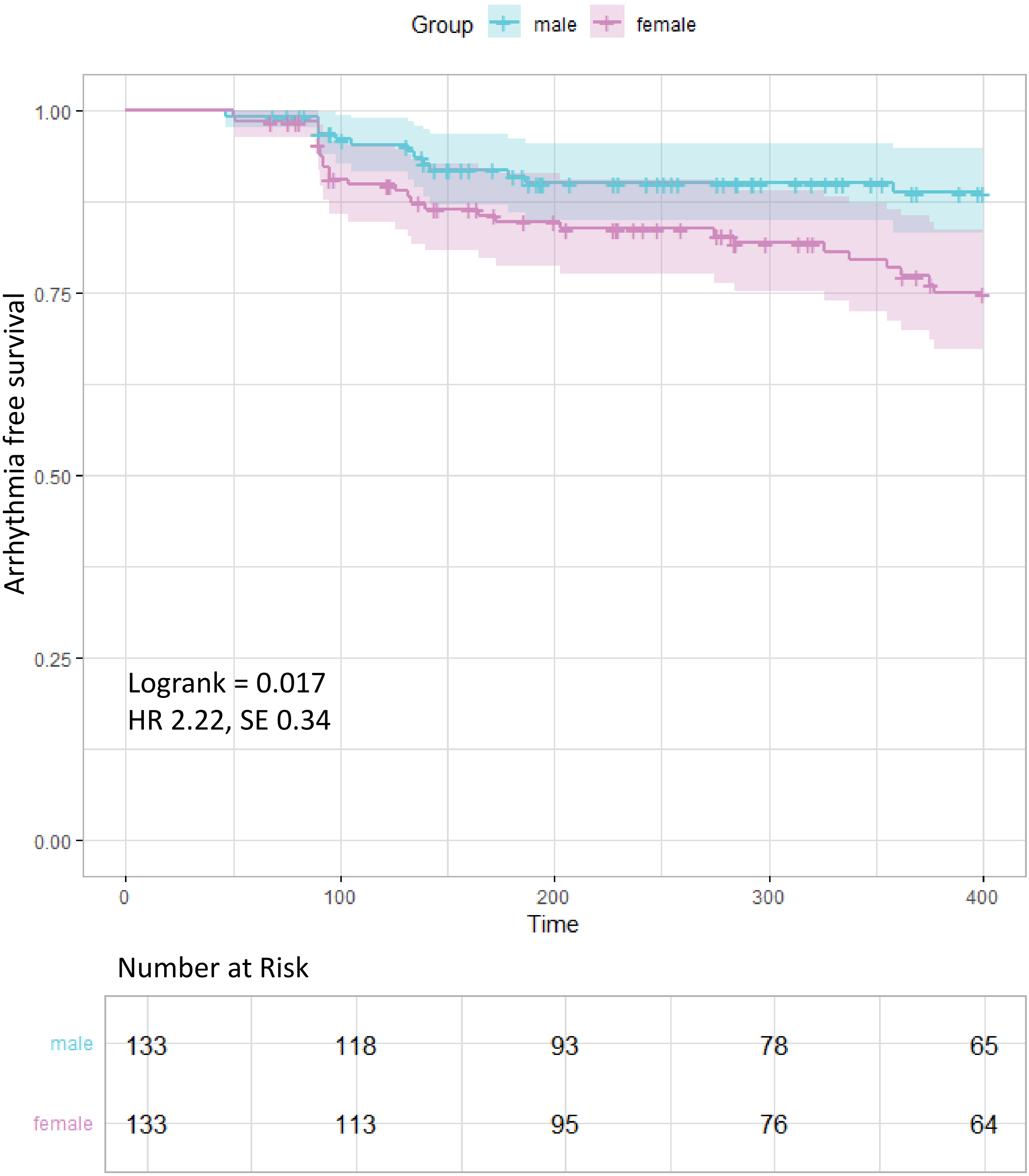

Supplement Figure 5: Kaplan Meier curve comparing female and male patients in the propensity matched cohort. The log rank test was used to determine the p-value. Time in days. Hazard Ratio (HR) female to male, Standard error (SE).
