# Supplementary material for: Sex differences in procedural characteristics, safety, and clinical outcomes of pulsed field ablation for atrial fibrillation
Source: Heart Rhythm O2. 2025 Oct 24;7(1):37–45. doi: 10.1016/j.hroo.2025.10.010 (PMC12902224; doi:10.1016/j.hroo.2025.10.010)
Supplement: Supplement Figure — 6 [file mmc6.pdf]

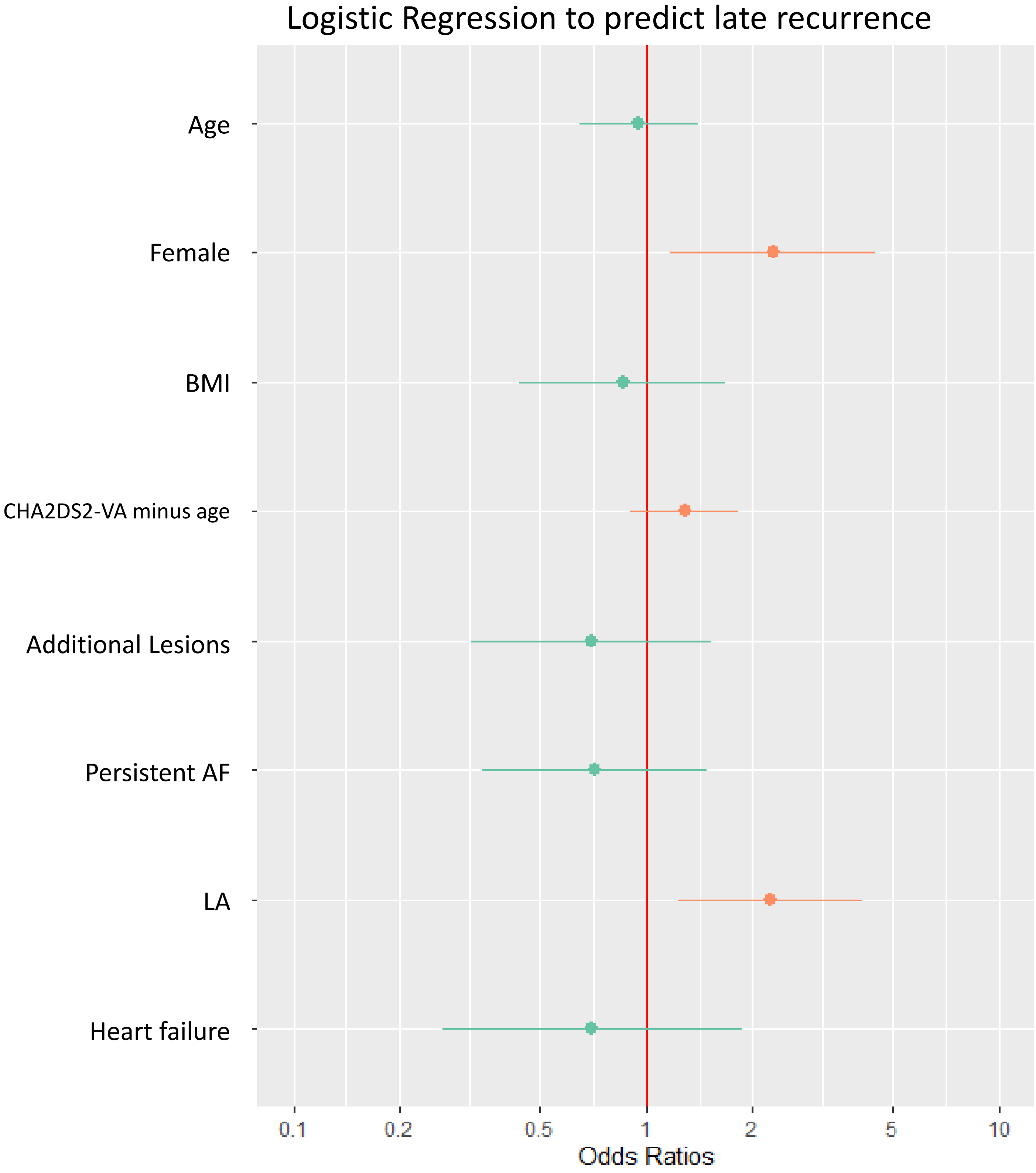

Supplement Figure 6: Forrest Plot for the Odds Ratios from the logistic regression model fitted to the propensity score matched population to identify potential predictors for atrial arrhythmia recurrence adjusted for the variables age (years), sex, BMI (kg/m2), CHA2DS2-VA score without age accounted for, if additional lesions were performed (binary, y/n), Atrial fibrillation type (binary, levels: persistent/paroxysmal), LA (left atrium diameter, mm) and history of heart failure (binary, y/n). Odds Ratios in Table format in Supplement Table 8.
