# Supplementary material for: Sex differences in procedural characteristics, safety, and clinical outcomes of pulsed field ablation for atrial fibrillation
Source: Heart Rhythm O2. 2025 Oct 24;7(1):37–45. doi: 10.1016/j.hroo.2025.10.010 (PMC12902224; doi:10.1016/j.hroo.2025.10.010)

Sex Differences in Procedural Characteristics, Safety and Clinical Outcomes of Pulsed-field Ablation for Atrial Fibrillation – Supplemental Materials

## Supplement Table 1: Patients with paroxysmal AF

| **Paroxysmal AF** | Overall | Female | Male | p |
| --- | --- | --- | --- | --- |
| n | 210 | 80 | 130 |  |
|  |  |  |  |  |
| Procedure time | 44 [32, 61] | 54 [40, 68] | 40 [29, 56] | <0.001 |
| LA time | 29 [19, 43] | 36 [23, 53] | 24 [16, 37] | <0.001 |
| Fluoroscopy time | 10 [8, 13] | 12 [8, 14] | 9 [7, 13] | 0.003 |
| Fluoroscopy dose | 359 [220, 660] | 345 [182, 631] | 362 [226, 674] | 0.279 |
| Total applications | 32 [26, 46] | 32 [26, 48] | 34 [26, 42] | 0.617 |
| PVI applications | 32 [19, 34] | 32 [18, 34] | 32 [19, 34] | 0.596 |
| Catheter size 35mm | 16 (7.6) | 4 (5.0) | 12 (9.2) | 0.393 |
|  |  |  |  |  |
| Additional lesions | 41 (19.5) | 18 (22.5) | 23 (17.7) | 0.5 |
| Mapping | 95 (45.2) | 45 (56.2) | 50 (38.5) | 0.018 |
| Hs-cTnT | 1626 [1077, 2155] | 1581 [1040, 2308] | 1626 [1092, 2101] | 0.758 |

Continuous variables expressed as ‘median [IQR]’, Factorial Variables as ‘absolute number (%)’. Abbreviations: LA Time, Left atrial dwell time; Fluoroscopy dose mcGy*m^2^; Times in minutes.

## Supplement Table 2: Patients with persistent AF

| **Persistent AF** | Overall | Female | Male | p |
| --- | --- | --- | --- | --- |
| n | 207 | 51 | 156 |  |
|  |  |  |  |  |
| Procedure time | 55 [43, 69] | 51 [40, 66] | 56 [45, 70] | 0.175 |
| LA time | 40 [27, 52] | 36 [24, 50] | 41 [30, 53] | 0.058 |
| Fluoroscopy time | 10 [8, 13] | 11 [8, 13] | 11 [8, 13] | 0.684 |
| Fluoroscopy dose | 479 [247, 904] | 377 [148, 618] | 538 [289, 957] | 0.001 |
| Total applications | 38 [28, 56] | 35 [24, 50] | 38 [28, 57] | 0.422 |
| PVI applications | 32 [18, 34] | 29 [18, 32] | 32 [18, 34] | 0.165 |
| Catheter size 35mm | 15 (7.2) | 4 (7.8) | 11 (7.1) | 1 |
|  |  |  |  |  |
| Additional lesions | 92 (44.4) | 22 (43.1) | 70 (44.9) | 0.957 |
| Mapping | 154 (74.4) | 32 (62.7) | 122 (78.2) | 0.044 |
| Hs-cTnT | 1214 [808, 1722] | 1094 [738, 1732] | 1220 [822, 1721] | 0.837 |

Continuous variables expressed as ‘median [IQR]’, Factorial Variables as ‘absolute number (%)’. Abbreviations: LA Time, Left atrial dwell time; Fluoroscopy dose mcGy*m^2^; Times in minutes.

## Supplement Table 3 Patients undergoing AF ablation

|  | Overall | paroxysmal | peristent | p |
| --- | --- | --- | --- | --- |
| n | 417 | 210 | 207 |  |
|  |  |  |  |  |
| Procedure time | 51 [38, 66] | 44 [32, 61] | 55 [43, 69] | <0.001 |
| LA time | 35 [22, 49] | 29 [19, 43] | 40 [27, 52] | <0.001 |
| Fluoroscopy time | 11 [8, 13] | 11 [8, 13] | 11 [8, 13] | 0.662 |
| Fluoroscopy dose | 389 [232, 783] | 359 [220, 660] | 479 [247, 904] | 0.007 |
| Total applications | 32 [19, 34] | 32 [19, 34] | 32 [19, 40] | 0.124 |
| PVI applications | 32 [18, 34] | 32 [19, 34] | 32 [18, 34] | 0.479 |
| CatheterSize 35mm | 31 (7.4) | 16 (7.6) | 15 (7.2) | 1 |
|  |  |  |  |  |
| Additional Lesions | 133 (31.9) | 41 (19.5) | 92 (44.4) | <0.001 |
| Hs-cTnT | 1412 [938, 1906] | 1626 [1077, 2155] | 1214 [808, 1722] | <0.001 |

Continuous variables expressed as ‘median [IQR]’, Factorial Variables as ‘absolute number (%)’. Abbreviations: LA Time, Left atrial dwell time; Fluoroscopy dose mcGy*m^2^; Times in minutes.

## Supplement Table 4: Patients undergoing AF ablation without 3D-EAM system

| Without EAM | Overall | Female | Male | p |
| --- | --- | --- | --- | --- |
| n | 171 | 56 | 115 |  |
|  |  |  |  |  |
| Procedure time | 35 [27, 46] | 41 [28, 53] | 34 [27, 44] | 0.126 |
| LA time | 21 [14, 30] | 23 [16, 32] | 20 [14, 27] | 0.142 |
| Fluoroscopy time | 9 [7, 13] | 11 [7, 13] | 9 [7, 11] | 0.086 |
| Fluoroscopy dose | 307 [154, 600] | 256 [104, 518] | 330 [203, 656] | 0.029 |
| Total applications | 32 [18, 34] | 32 [18, 34] | 32 [19, 34] | 0.6 |
| PVI applications | 32 [18, 34] | 32 [18, 34] | 32 [19, 34] | 0.691 |
| Catheter size 35mm | 24 (14.0) | 7 (12.5) | 17 (14.8) | 0.866 |
|  |  |  |  |  |
| Additional lesions | 24 (14.0) | 5 (8.9) | 19 (16.5) | 0.268 |
| Hs-cTnT | 1525 [1046, 2038] | 1298 [998, 2089] | 1598 [1095, 1962] | 0.2 |

Continuous variables expressed as ‘median [IQR]’, Factorial Variables as ‘absolute number (%)’. Abbreviations: LA Time, Left atrial dwell time; Fluoroscopy dose mcGy*m^2^; Times in minutes.

## Supplement Table 5: Patients undergoing AF ablation with 3D-EAM system

| With EAM | Overall | Female | Male | p |
| --- | --- | --- | --- | --- |
| n | 254 | 78 | 176 |  |
|  |  |  |  |  |
| Procedure time | 60 [48, 71] | 62 [48, 71] | 59 [48, 71] | 0.668 |
| LA time | 42 [33, 54] | 42 [33, 54] | 42 [32, 53] | 0.692 |
| Fluoroscopy time | 11 [8, 14] | 12 [9, 14] | 11 [8, 14] | 0.074 |
| Fluoroscopy dose | 479 [269, 894] | 395 [246, 672] | 538 [296, 986] | 0.005 |
| Total applications | 32 [24, 40] | 32 [18, 38] | 32 [26, 40] | 0.438 |
| PVI applications | 32 [18, 34] | 32 [18, 32] | 32 [18, 34] | 0.38 |
| Catheter size 35mm | 8 (3.1) | 2 (2.6) | 6 (3.4) | 1 |
|  |  |  |  |  |
| Additional lesions | 113 (44.5) | 36 (46.2) | 77 (43.8) | 0.827 |
| Hs-cTnT | 1340 [843, 1850] | 1541 [926, 2190] | 1305 [834, 1756] | 0.031 |

Continuous variables expressed as ‘median [IQR]’, Factorial Variables as ‘absolute number (%)’. Abbreviations: LA Time, Left atrial dwell time; Fluoroscopy dose mcGy*m^2^; Times in minutes.

## Supplement Table 6: Log Regression Odds Ratios

|  | OR | Low CI | High CI | p |
| --- | --- | --- | --- | --- |
| Age | 0.87 | 0.66 | 1.16 | 0.359 |
| Female | 2.17 | 1.23 | 3.81 | 0.007 |
| BMI | 0.76 | 0.44 | 1.34 | 0.350 |
| CHA2DS2-VA minus age | 1.32 | 1.01 | 1.73 | 0.041 |
| Additional Lesions | 0.53 | 0.28 | 0.99 | 0.045 |
| Persistent AF | 0.87 | 0.50 | 1.52 | 0.625 |
| LA | 1.80 | 1.18 | 2.76 | 0.006 |
| Heart failure | 0.89 | 0.42 | 1.87 | 0.751 |

Odds Ratios (OR) of the Logistic regression model with lower 95% Confidence Interval (Low CI), high 95% Confidence Interval (High CI) and p-Value. Variables used were Age/10, Female sex, BMI/10, CHA2DS2-VA minus age, Additional Lesions, Persistent AF, LA/10 and Heart Failure.

## Supplement Table 7: Baseline characteristics for the propensity score-matched cohort

|  | Overall | Female | Male | p |
| --- | --- | --- | --- | --- |
| n | 266 | 133 | 133 |  |
|  |  |  |  |  |
| Age | 69 [62, 74] | 70 [62, 75] | 69 [62, 73] | 0.224 |
| BMI | 26.1 [23.4, 29.6] | 25.3 [23.0, 29.4] | 26.5 [23.8, 29.6] | 0.256 |
|  |  |  |  |  |
| Paroxysmal AF | 152 (57.1) | 79 (59.4) | 73 (54.9) | 0.536 |
| Persistent AF | 109 (41.0) | 51 (38.3) | 58 (43.6) | 0.454 |
| Diabetes | 26 (9.8) | 13 (9.8) | 13 (9.8) | 1 |
| Hypertonie | 153 (57.5) | 77 (57.9) | 76 (57.1) | 1 |
| CAD | 11 (4.1) | 8 (6.0) | 3 (2.3) | 0.218 |
| Heart failure | 44 (16.5) | 24 (18.0) | 20 (15.0) | 0.621 |
| Stroke | 7 (2.6) | 3 (2.3) | 4 (3.0) | 1 |
|  |  |  |  |  |
| LA | 39 [35, 43] | 39 [35, 43] | 40 [35, 43] | 0.638 |
| LAVI | 37 [30, 45] | 39 [29, 47] | 36 [30, 42] | 0.154 |
| LVEF | 59 [51, 63] | 60 [53, 64] | 56 [50, 62] | 0.031 |
| Septum | 10 [8, 11] | 9 [8, 10] | 10 [9, 12] | 0.001 |
| Posterior Wall | 9 [8, 10] | 9 [7, 10] | 9 [8, 10] | <0.001 |
|  |  |  |  |  |
| AAD | 110 (41.4) | 58 (43.6) | 52 (39.1) | 0.534 |
| Betablocker | 193 (72.6) | 101 (75.9) | 92 (69.2) | 0.272 |
| Vit K Anta | 12 (4.9) | 6 (4.8) | 6 (4.8) | 1 |
| DOAC | 226 (85.0) | 117 (88.0) | 111 (83.5) | 1 |
|  |  |  |  |  |
| ILR | 30 (11.3) | 8 (6.0) | 22 (16.5) | 0.012 |

Continuous variables expressed as ‘median [IQR]’, Factorial Variables as ‘absolute number (%)’. Abbreviations and Units: Age, years; BMI, Body mass Index, kg/m^2^; AF, Atrial fibrillation; CAD, coronary artery disease; LAVI, left atrial volume index, ml/m2; LVEF, Left ventricular ejection fraction, %; Septum, Septum Thickness, mm; Posterior Wall, Posterior Wall thickness, mm; AAD, Anti-Arhythmic Drugs; Vit K Anta, Vitamin K Antagonist (i.e. Marcoumar); DOAC, Direct oral anticoagulation; ILR, Implantable loop recorder

## Supplement Table 8: Log Regression Odds Ratios for the Propensity Score-matched cohort

|  | OR | Low CI | High CI | p |
| --- | --- | --- | --- | --- |
| Age | 0.95 | 0.65 | 1.41 | 0.813 |
| Female | 2.28 | 1.16 | 4.48 | 0.016 |
| BMI | 0.86 | 0.44 | 1.67 | 0.647 |
| CHA2DS2-VA minus age | 1.28 | 0.90 | 1.82 | 0.169 |
| Additional Lesions | 0.70 | 0.32 | 1.54 | 0.375 |
| Persistent AF | 0.71 | 0.34 | 1.48 | 0.365 |
| LA | 2.24 | 1.23 | 4.07 | 0.008 |
| Heart failure | 0.70 | 0.26 | 1.86 | 0.477 |

Odds Ratios (OR) of the Logistic regression model with lower 95% Confidence Interval (Low CI), high 95% Confidence Interval (High CI) and p-Value. Variables used were Age/10, Female sex, BMI/10, CHA2DS2-VA minus age, Additional Lesions, Persistent AF, LA/10 and Heart Failure.

## Supplement Figure 1

Kaplan Meier curve comparing female and male patients with paroxysmal atrial fibrillation. The log rank test was used to determine the p-value. Time in days. Hazard Ratio (HR) female to male, Standard error (SE).


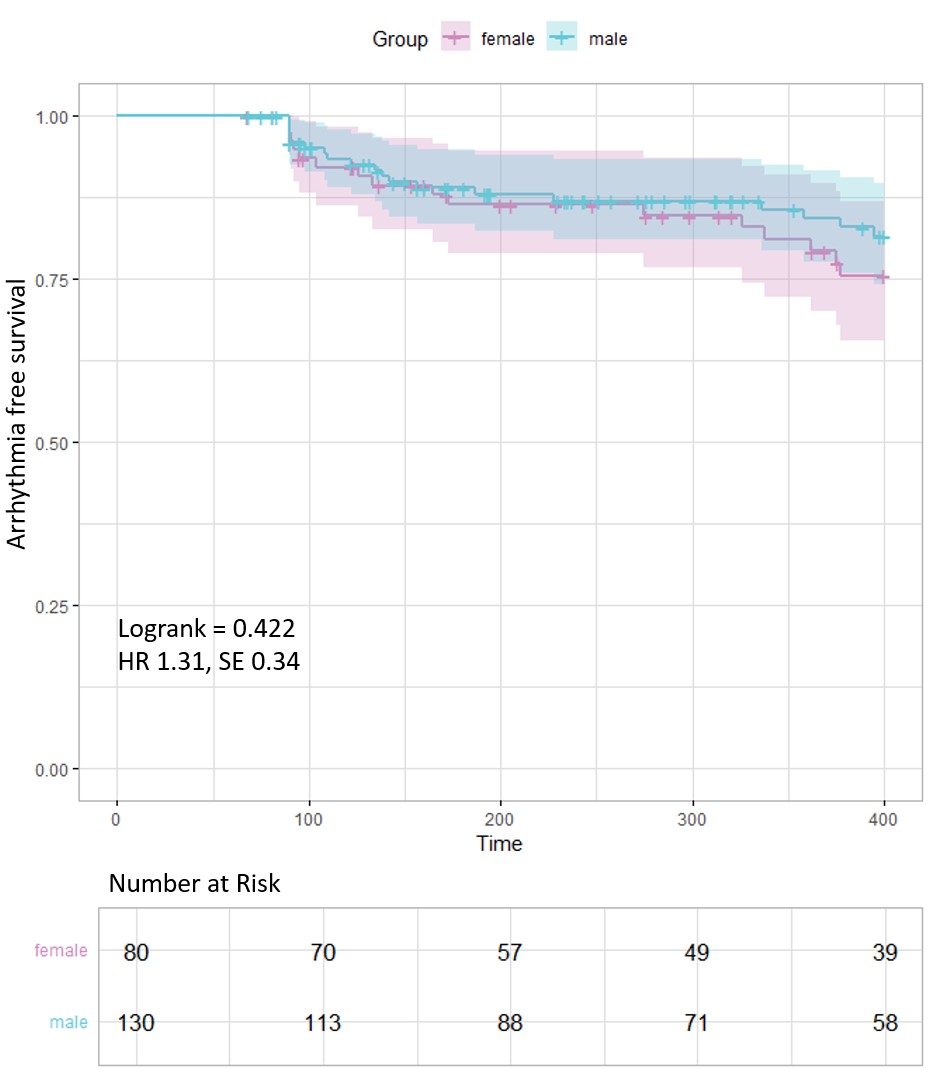


## Supplement Figure 2

Kaplan Meier curve comparing female and male patients with persistent atrial fibrillation. The log rank test was used to determine the p-value. Time in days. Hazard Ratio (HR) female to male, Standard error (SE).
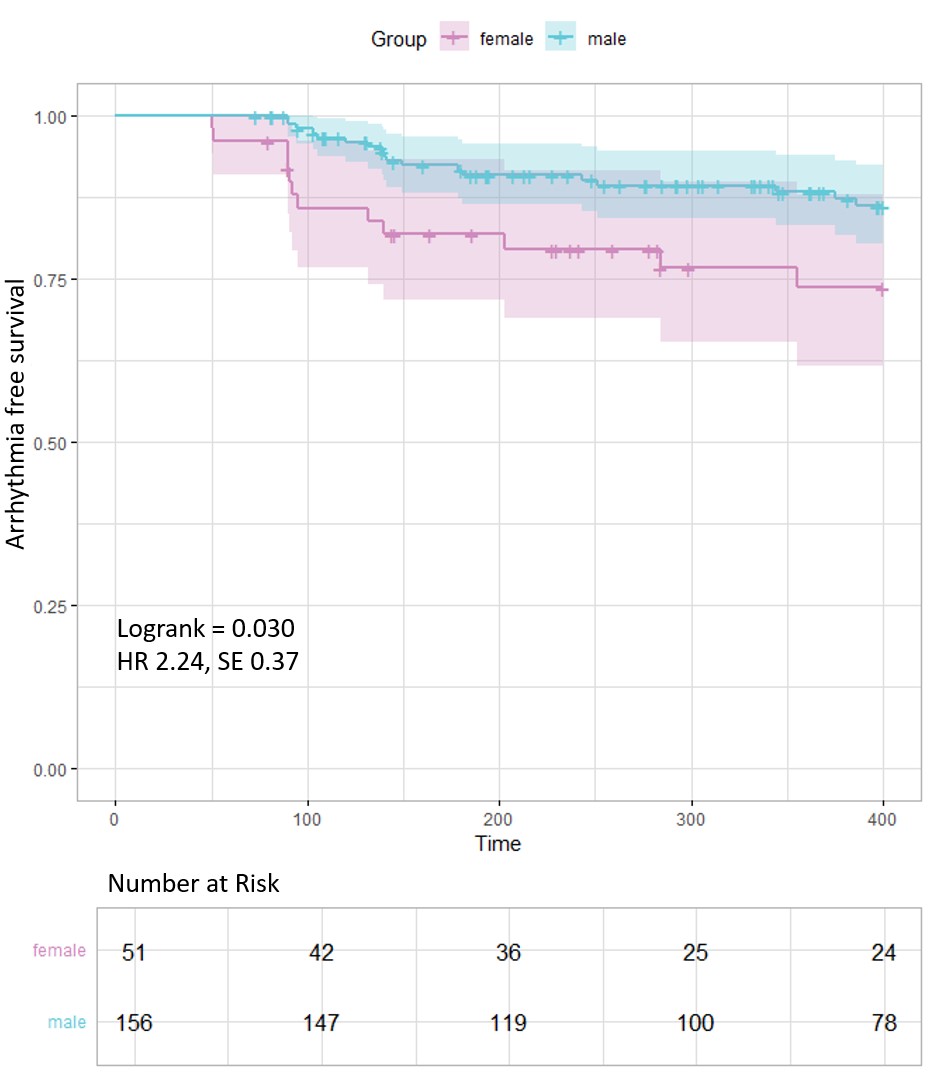


## Supplement Figure 3

Kaplan Meier curve comparing patients with paroxysmal atrial fibrillation vs persistent atrial fibrillation in the overall population. The log rank test was used to determine the p-value. Time in days. Hazard Ratio (HR) persistent to paroxysmal, Standard error (SE)


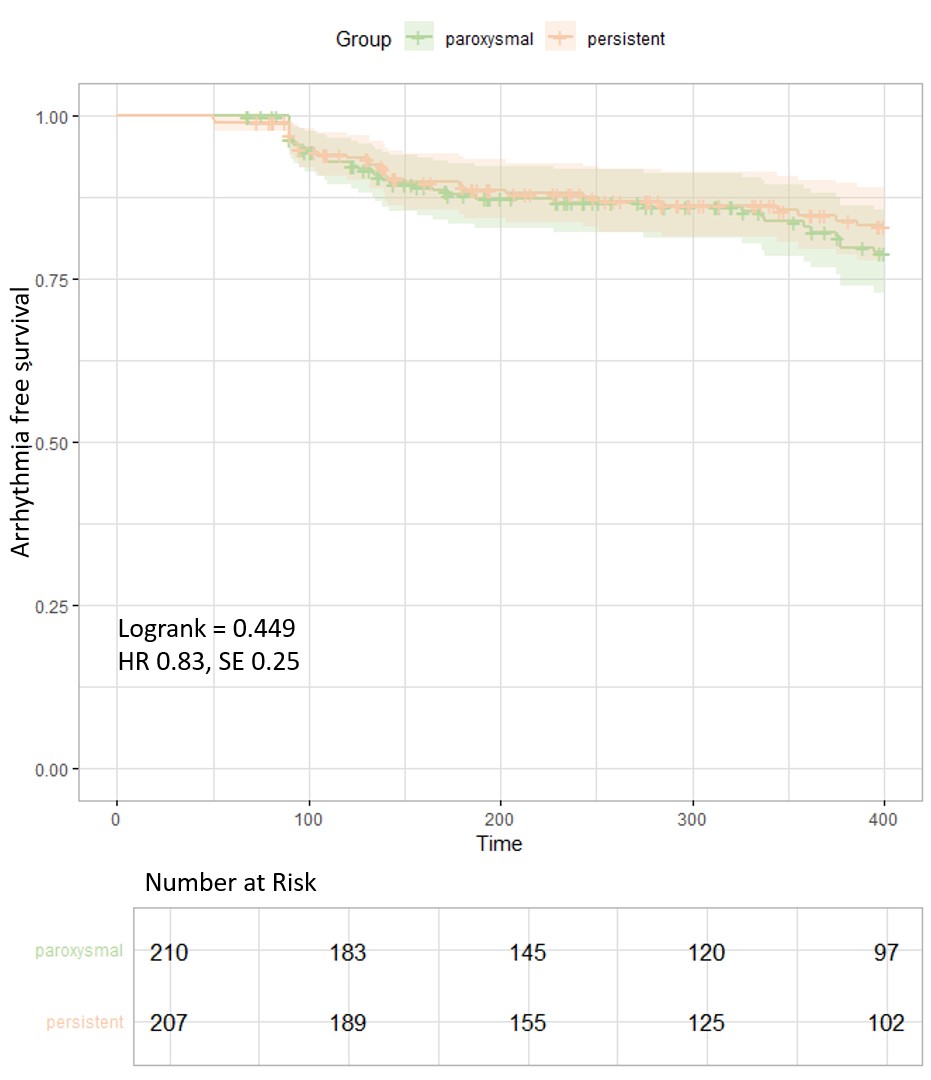


## Supplement Figure 4

Kaplan Meier curve comparing female and male patients with PVI-only approach. The log rank test was used to determine the p-value. Time in days. Hazard Ratio (HR) female to male, Standard error (SE).
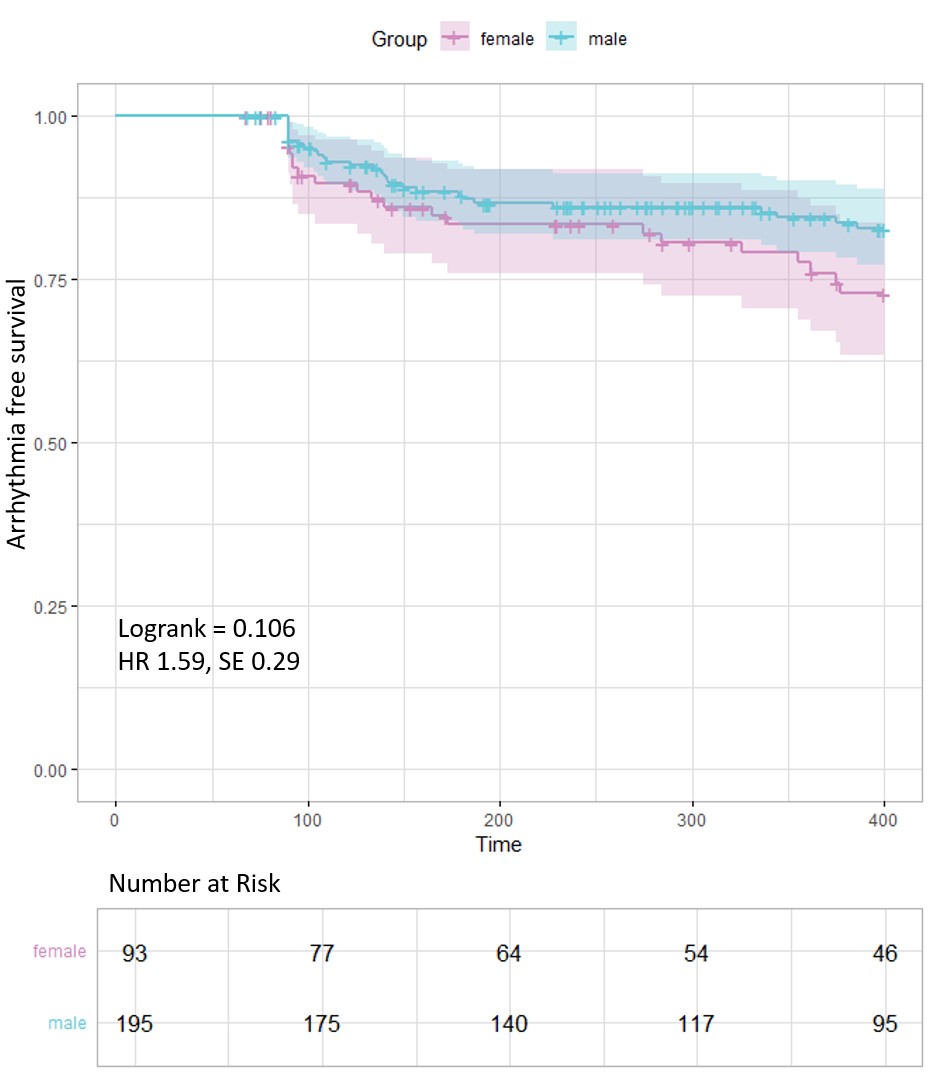


## Supplement Figure 5

Kaplan Meier curve comparing female and male patients in the propensity matched cohort. The log rank test was used to determine the p-value. Time in days. Hazard Ratio (HR) female to male, Standard error (SE).


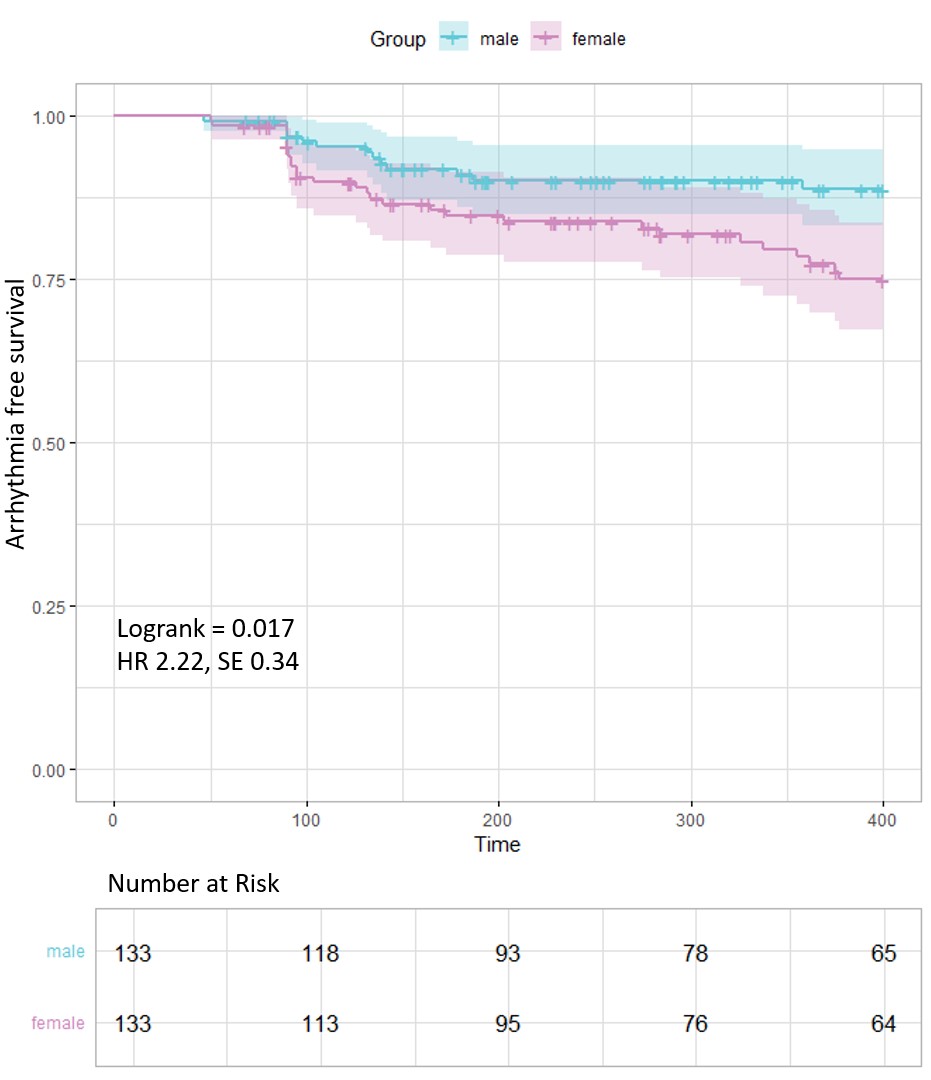


## Supplement Figure 6

Forrest Plot for the Odds Ratios from the logistic regression model fitted to the propensity score matched population to identify potential predictors for atrial arrhythmia recurrence adjusted for the variables age (years), sex, BMI (kg/m^2^), CHA2DS2-VA score without age accounted for, if additional lesions were performed (binary, y/n), Atrial fibrillation type (binary, levels: persistent/paroxysmal), LA (left atrium diameter, mm) and history of heart failure (binary, y/n). Odds Ratios in Table format in Supplement Table 8.


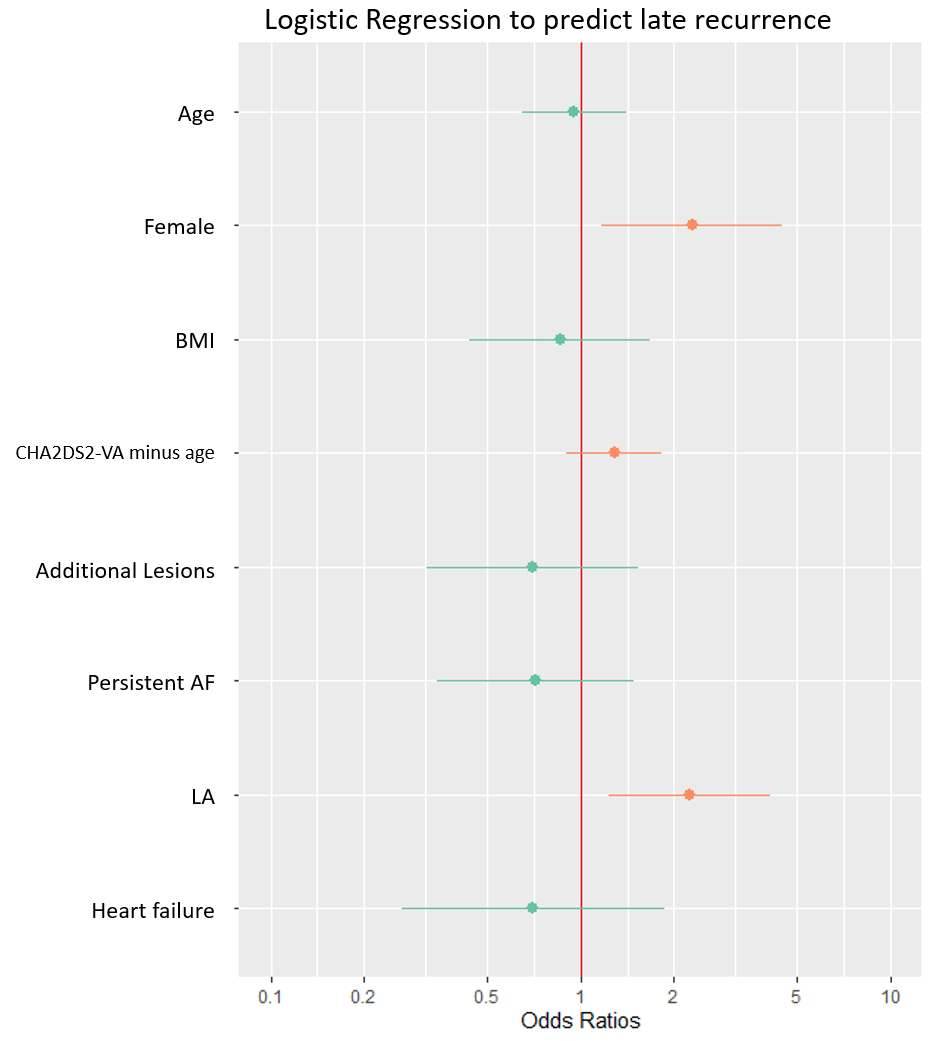

Supplement: Supplemental Materials [file mmc7.docx]
